# Urinary polycyclic aromatic hydrocarbon metabolites and their association with oxidative stress among pregnant women in Los Angeles

**DOI:** 10.21203/rs.3.rs-4119505/v1

**Published:** 2024-03-20

**Authors:** Qi Meng, Sanjali Mitra, Irish Del Rosario, Michael Jerrett, Carla Janzen, Sherin U. Devaskar, Beate Ritz

**Affiliations:** University of California, Los Angeles; University of California, Los Angeles; University of California, Los Angeles; University of California, Los Angeles; University of California, Los Angeles; University of California, Los Angeles; University of California, Los Angeles

**Keywords:** PAH (polycyclic aromatic hydrocarbons), Oxidative stress, Pregnancy

## Abstract

**Background:**

Polycyclic aromatic hydrocarbons (PAHs) have been linked to adverse birth outcomes, but few epidemiological studies to date have evaluated associations between urinary PAH metabolites and oxidative stress biomarkers in pregnancy.

**Methods:**

We measured a total of 7 PAH metabolites and 2 oxidative stress biomarkers (malondialdehyde (MDA), 8-hydroxy-2’-deoxyguanosine (8-OHdG)) in urine samples collected up to three times during pregnancy in 159 women enrolled at antenatal clinics at the University of California Los Angeles during 2016–2019. Using multiple linear regression models, we estimated the percentage change (%) and 95% confidence interval (CI) in 8-OHdG and MDA measured at each sample collection time per doubling of PAH metabolite concentrations.

**Results:**

Most PAH metabolites were positively associated with both urinary oxidative stress biomarkers, MDA and 8-OHdG, with stronger associations in early and late pregnancy. Women pregnant with male fetuses exhibited slightly larger increases in both MDA and 8-OHdG in association with PAH exposures in early and late pregnancy.

**Conclusion:**

Urinary OH-PAH biomarkers are associated with increases in oxidative stress during pregnancy, especially in early and late pregnancy. Sex differences in associations between PAH exposures and oxidative stress need to be further explored in the future.

## 1 Background

Polycyclic aromatic hydrocarbons (PAHs) are widespread environmental pollutants resulting from incomplete combustion or pyrolysis of organic matter [1]. Due to their ubiquity and toxicity, PAHs are of global public health concern [2]. The sources of human exposures to PAHs are diverse and include tobacco smoke, industrial sites, wildfires, residential wood fires, ambient air pollution, and dietary items such a charbroiled meat [1, 3]. Some PAHs, such as Benzo[a]pyrene and Benzo[b] fluoranthene, have been listed as carcinogens by IARC [4]. Moreover, PAHs can act as endocrine disrupters [5].

Prenatal exposures to PAHs have been linked to adverse birth outcomes such as fetal growth restriction and preterm birth [6, 7]. They have also been related to neurodevelopment [8] and childhood asthma [9], yet the biological mechanisms through which PAHs exhibit such toxicity are still debated. PAHs have been reported to induce oxidative stress, inflammation and endocrine disruptions, which may each individually or jointly have adverse effects on the pregnancy [10–12]. Specifically, PAHs can induce an excess of reactive oxygen species (ROS), which would target DNA, proteins and lipids, resulting in their oxidation [12]. Well-established biomarkers of oxidative stress include products of oxidation processes such as malondialdehyde (MDA) and 8-hydroxy-2’-deoxyguanosine (8-OHdG), which represents lipid peroxidation and oxidative DNA damage, respectively [13].

Urinary concentrations of hydroxylated metabolites of PAHs (OH-PAHs) have been widely used as biomarkers of PAH exposure, as they represent the integrated levels of exposure across multiple pathways in the hours or days prior to sampling [14]. Nevertheless, few studies up to date used urinary PAH biomarkers to examine the effect of PAH exposures on oxidative stress levels among pregnant women. A cohort study of 200 pregnant women in Boston collected urine samples in the 3rd trimester of pregnancy and found increases in urinary 8-hydroxydeoxyguanosine (8-OHdG), among women with higher concentrations of the PAH metabolite, 2-hydroxynapthalene (2-NAP) [15]. Similarly, several PAH metabolites including 2-NAP, fluorene, and phenanthrene were reported to be correlated with urinary concentrations of 8-OHdG in a cohort of 188 pregnant Chinese women at different times in pregnancy [16]. Finally, a larger study of 715 South Korean pregnant women also reported that urinary concentrations of 2-NAP and pyrene were associated with increases in MDA at 12–28 weeks of gestation [17]. These studies, however, did not collect multiple samples during pregnancy and, thus, could not control for intra-individual variability in urinary PAH metabolites. Some also focused on a specific gestational window but none could address whether critical windows exist in which PAH exposures increase oxidative stress during pregnancy.

Here, we collected urine samples from pregnant women enrolled at UCLA antenatal clinics for up to three times during pregnancy and evaluated associations between urinary PAH metabolite concentrations and oxidative stress biomarkers in different gestational periods during pregnancy.

## 2 Methods

### 2.1 Study population

The Imaging Innovations for Placental Assessment in Response to Environmental Exposures (PARENTs) study recruited a cohort of 199 women early in pregnancy from antenatal clinics at the University of California Los Angeles during 2016–2019 [18]. Women were enrolled as early as the 10th week of gestation and were asked to participate in a once-per-trimester and at-birth study visit that included urinary sample collections we used for oxidative stress biomarkers and metabolites of hydroxyl polycyclic aromatic hydrocarbons (OH-PAHs) assessment. Phone interviews were conducted at three timepoints during pregnancy and at birth to collect environmental and behavioral risk factor data.

Our study population consists of 159 women enrolled in the PARENTs study for whom at least one useable urine sample was available at the time of laboratory analysis supported by the Emory Children’s Health Exposure Analysis Resource (CHEAR) program.

### 2.2 Biomarkers assessment

We focused on two oxidative stress biomarkers, malondialdehyde (MDA) and 8-hydroxy-2’-deoxyguanosine (8-OHdG), and a total of 7 hydroxyl PAH metabolites (OH-PAHs). Specifically, we examined measures for the combined 2-hydroxyfluorene + 3-hydroxy fluorene (2&3-FLUO) metabolites, the single metabolites 2-hydroxynaphthalene (2-NAP), 1-hydroxyphenanthrene (1-PHEN), 2-hydroxyphenanthrene (2-PHEN), 3-hydroxyphenanthrene (3-PHEN), 4-hydroxyphenanthrene (4-PHEN), and 1-hydroxypyrene (1-PYR), and also the sum of 1-, 2-, 3-, and 4- hydroxyphenanthrene (Σ_4_OH-PHEN), and of all 7 analytes (Σ_7_OH-PAH), respectively.

Study visits were timed to be optimal for Magnetic resonance imaging (MRI) evaluations (1st MRI 14th-18th gestational week, 2nd MRI 19th −24th gestational week) in the PARENTs cohort study, and sample collection followed this schedule with the 1st sample being collected in the 10–17th gestational week, the 2nd sample collection in the 18–29th gestational week, and the 3rd sample collection after the 30th gestational week and prior to delivery. Maternal urinary samples were collected at each study visit, and we collected at least one and at most three urine samples from all participants during pregnancy. Specifically, multiple urine samples were collected among 146 out of 159 participants (92%).

The urine samples were stored at −80°C after collection at UCLA and were shipped on dry ice to the CHEAR Laboratory Hub to measure both the OH-PAHs and the oxidative stress biomarkers. All samples were randomized using a Fisher-Yates shuffling algorithm prior to laboratory analysis to reduce any potential batch effects [19, 20]. The OH-PAHs were measured by tandem mass spectrometry (MS/MS) [21], and the oxidative stress biomarkers (MDA and 8-OHdG) were measured by liquid chromatography-mass spectrometry (LC-MS) [22].

Samples with measures below the limit of detection (LOD) values were replaced with the $\text{LOD}/\sqrt{2}$ [23]. Concentrations were further corrected for urine dilution by adjusting for specific gravity (SG) measured with a Reichert AR200 refractometer. We excluded samples with an invalid SG value below 1 (N = 14, 18 and 16 samples during the 1st, 2nd and 3rd sample collection interval, respectively), resulting in a total of 391 samples available for analysis. To correct for the hydration status of pregnant women, SG-standardized biomarker concentrations were calculated using the following formula [24]:

$$CHEM_{SG\_Adj}=CHEM_{i}*\left[\left(SG_{m}-1\right)/\left(SG_{i}-1\right)\right]$$

where CHEM_SG_Adj_ is the specific gravity-standardized biomarker concentration (nmol/L for MDA, ng/mL for 8-OHdG, ng/L for OH-PAHs), CHEM_i_ is the observed biomarker concentration, SG_i_ is the specific gravity of the urine sample and SG_m_ is the median specific gravity for the total samples with valid SG values.

### 2.3 Covariates

Information on maternal age (years), parity (continuous), maternal pre-pregnancy body mass index (BMI) (< 18.5, 18.5–24.9, 25.0–30.0 and ≥ 30.0), maternal race/ethnicity (White, non-White), smoking (yes, no), maternal educational attainment (bachelor’s degree or less, master’s degree, doctoral/professional degree) were collected in interviews. Gestational age (based on the best obstetric estimate obtained during a 1st trimester ultrasound exam), as well as information about pregnancy complications including gestational diabetes, gestational hypertension, and pre-eclampsia were obtained from medical records. Season of sample collection was categorized based on the month of sample collection, as spring (March, April, May), summer (June, July, August), fall (September, October, November) and winter (December, January, February).

### 2.4 Statistical analysis

First, we estimated effects for different time intervals during pregnancy by first conducting multiple linear regression analyses and calculated the expected percentage of change in each oxidative stress biomarker concentration according to PAH metabolite levels in each sample collection interval, separately. The oxidative stress biomarker concentrations were treated as continuous variables and log-transformed for statistical analyses. For all OH-PAHs, we log-transformed (base 2) the values such that in statistical model the exposure effect estimate represents an increase per doubling of the OH-PAHs concentration (ng/L). Second, we also used linear mixed models with a random intercept for participant to take repeated measurements into account, i.e., we relied on up to 3 samples collected across pregnancy for each exposure and biomarker and assessed associations between OH-PAHs exposure urine measures and oxidative stress concentrations across multiple time points in pregnancy.

In one-point-in-time linear and in longitudinal linear mixed regression models, we adjusted for maternal age, maternal race/ethnicity, maternal education, parity, pre-pregnancy BMI, and season of sampling. We also conducted stratified one-point-in-time linear regression analyses by season of sampling, fetal sex, and maternal race/ethnicity to evaluate potential effect measure modification. Sensitivity analyses were conducted by additionally adjusting for gestational day at sample collection. Furthermore, as women experiencing pregnancy complications are likely to have higher oxidative stress levels due to these conditions [25], we also restricted some analyses to women without pregnancy complications Specifically gestational diabetes, gestational hypertension, or pre-eclampsia. All statistical analyses were performed using SAS 9.4 (SAS Institute Inc., Cary, NC, USA).

## 3 Results

Most of our study participants were ≥ 30 years old and highly educated, more than half were parous, and almost a third (31%) were overweight or obese (BMI ≥ 25) (Table 1). Approximately half of the mothers reported their race/ethnicity as non-Hispanic White; 18% as Hispanic and 28% as Asian/Pacific Islander. The demographics characteristics of the entire PARENTs cohort are very similar to those of the subpopulation used in this study (Table S1).

The average MDA concentrations decreased from the 1st to 2nd sample collection and increased in the 3rd time interval (Table S2). The concentrations of 8-OHdG decreased throughout the three sampling periods over pregnancy. While the hydroxy PAH metabolites, 2-NAP and 2-PYR levels, increased throughout the three sampling periods, the 2&3-FLUO and all OH-PHEN isomers decreased slightly from the 1st to 2nd sample collection but increased again at the 3rd sample collection time. For both oxidative stress biomarkers we measured the lowest average concentrations in summer and relatively higher concentrations in fall and winter (Table S3). For the PAH metabolites including 2-NAP and 1-PYR we also saw relatively higher concentration levels in fall and winter, while 2&3-FLUO and all the OH-PHEN isomers increased from spring to summer, then decreased in fall and increased again in winter, thus exhibiting relatively higher levels in summer and in winter. In each pregnancy sample collection period, the two oxidative stress biomarkers were highly correlated with each other (r = 0.8) as were the OH-PHEN isomers and 1-PYR (r = 0.6–0.9) (Figure S1), and the OH-PHEN isomers were moderately to highly correlated with the two oxidative stress biomarkers, except in the 2nd pregnancy sampling period.

**Figure 1:**
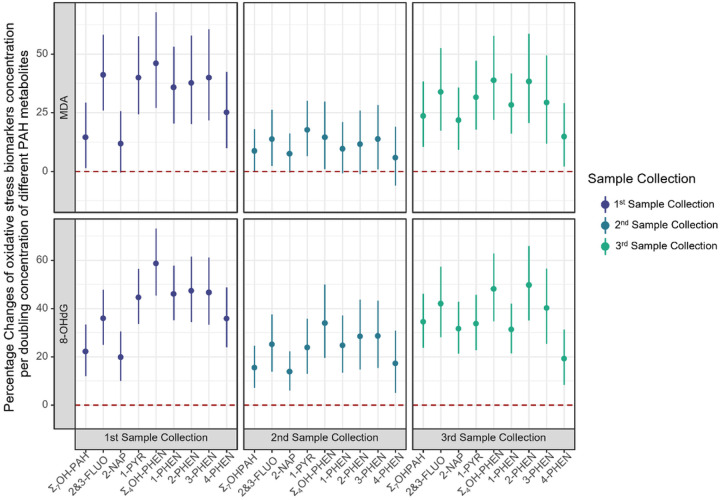
**Linear regression for percentage changes of oxidative stress biomarker concentrations per doubling concentration of different PAH metabolites**^a^. a. Adjusted for maternal age, maternal race/ethnicity, maternal education, pre-pregnancy BMI, and sampling season.

In one-pregnancy period at a time linear regression models, a doubling of each urinary PAH metabolite concentration increased the MDA (a lipid peroxidation marker) concentrations by 5.8%−41.1%, with the lowest effects estimated in the 2nd sampling period (Fig. 1; Table S4), and some of the 95% CIs including the null value. MDA concentration also increased by 8.7%−23.6% in each of the three sampling periods with a doubling of the Σ_7_OH-PAH. Specifically, a doubling of 2&3-FLUO concentrations increased MDA levels by 13.7%−41.1% and of 1-PYR by 17.7%−39.9%. 2-NAP concentrations were associated with a 21.8% (95% CI: 9.2%, 35.8%) increase in MDA measured in the 3rd sampling period, and were also increased in the first two periods, but the 95% CIs included the null. MDA increases ranged from 14.5–46.0% per doubling in Σ_4_OH-PHEN; and each individual OH-PHEN isomer exhibited a similar pattern as the summary measure, with the smallest effects estimated for 4-PHEN.

Positive associations were also observed for 8-OHdG (a DNA damage marker) with each PAH exposure in simple one-pregnancy period only linear regression models. Although effect estimates were smaller in the 2nd gestational sampling period, most of the 95% CIs overlapped for the three sampling periods (Fig. 1; Table S4). A doubling of Σ_7_OH-PAH concentration was associated with a 22.2%, 15.5% and 34.5% increase in urinary 8-OHdG in the 1st, 2nd and 3rd sampling period, respectively. In all three sampling periods, concentrations of 8-OHdG increased with a doubling in urinary concentrations of 2&3-FLUO by 25.1%−42.0%, 2-NAP by 13.8%−31.6%, and 1-PYR by 23.8%−44.6%, respectively. Σ_4_OH-PHEN were also found to increase 8-OHdG in all three sampling periods by 33.9%−58.6%; and similar to MDA, smaller effect estimates were observed for 4-PHEN.

**Figure 2:**
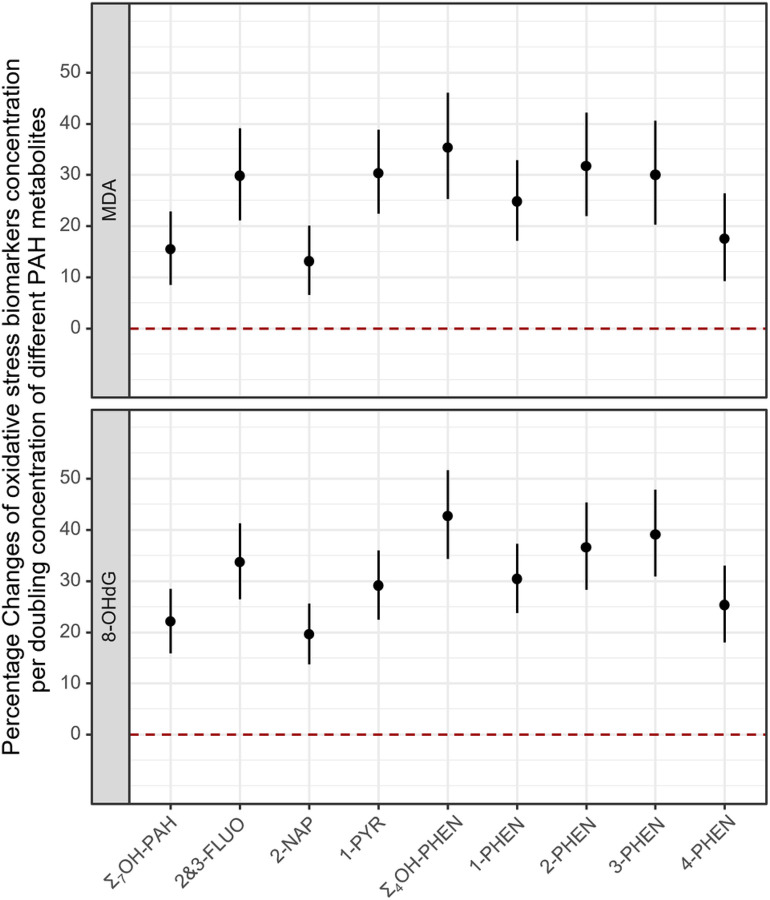
**Linear mixed model for percentage changes of oxidative stress biomarker concentrations per doubling concentration of different PAH metabolites**^a^. a. Adjusted for maternal age, maternal race/ethnicity, maternal education, pre-pregnancy BMI, and sampling season.

Linear mixed models that included all three pregnancy sample collection periods showed consistent patterns with those from separate linear regression models (Fig. 2; Table S5). We estimated a 15.5% (95% CI: 8.5%, 22.9%) increase of MDA levels and a 22.1% (95% CI: 15.9%, 28.5%) increase of 8-OHdG levels associated with a doubling of Σ_7_OH-PAH concentration. The effect estimates for specific PAH metabolites ranged from 13.1–30.3% for MDA concentrations, and from 19.6–39.1% for 8-OHdG concentrations, with somewhat less strong associations for 2-NAP and 4-PHEN.

When stratifying by sampling season in simple one-pregnancy sampling period linear regression models, stronger effect estimates were generally observed in fall and winter for most PAH exposures and both oxidative stress biomarkers, especially for the 3rd gestational period (Figure S2). When stratifying by fetal sex, we estimated slightly larger effect estimates among male fetuses for most PAH exposures and lipid peroxidation or DNA damage in early and late pregnancy, although the 95% CIs for male and female fetuses generally overlapped especially for MDA in the 3rd sampling period (Figure S3). Compared to non-White women, we saw that for White women PAH exposures were associated with higher oxidative stress biomarker levels in the 1st sampling period, except for 2&3-FLUO and 8-OHdG (Figure S4).

Finally, in one-pregnancy-time-point linear regression, effect estimates slightly increased after restricting to women without pregnancy complications (Table S6). Results changed minimally after additionally adjusting for gestational days at sample collection date (data not shown).

## 4 Discussion

In our study of pregnant women, we found consistent associations between several urinary PAH metabolites and two oxidative stress biomarkers for lipid peroxidation, MDA, and DNA damage, 8-OHdG. Stronger associations were seen in early and late pregnancy, especially for urine samples collected in the fall or winter season. Effect estimates in different gestational period also differed by fetal sex with slightly stronger associations seen in early and late pregnancy in mothers carrying male fetuses. To our knowledge, this is the first longitudinal study with repeated urinary measures to assess associations between urinary PAH metabolites and two oxidative stress biomarkers during pregnancy. This also allowed us to additionally investigate whether there are susceptible windows for PAH exposures across pregnancy and according to fetal sex.

The metabolism of PAHs depends on the cytochrome P450-mediated mixed function oxidase system that produces enormous quantities of reactive intermediates involved in lipid peroxidation, protein modification, DNA damage, and the depletion of endogenous antioxidants [26]. Our findings for PAH metabolites including 2&3-FLUO, 2-NAP, 1-PYR, and OH-PHENs being associated with increases in urinary 8-OHdG and MDA during pregnancy are consistent with the few previously published reports investigating this subject [15–17]. However, studies in pregnant women that investigate associations between PAH exposures and oxidative stress in different time periods during pregnancy are rare. The stronger associations we observed in early and late pregnancy were consistent with the sensitive window for adverse birth outcomes such as preterm birth and term low birth weight, which have been widely reported to be associated with prenatal exposures to environmental pollutants and the oxidative stress they induce [27–30].

Stronger associations between PAH exposures and oxidative stress biomarkers have also been detected in the winter season, which may be explained by seasonally higher atmospheric PAH concentrations in winter than summer for most compounds due to an increased production from heating related combustion and adverse meteorological conditions [31], suggesting higher toxicity of the mixture from combustion sources. Apart from smoking and ambient or indoor air pollution from traffic and combustion including open replaces and wood burning, dietary intake is one of the main sources of PAH exposures in the general population, including food contamination and food processing procedures such as smoking, drying, and frying of foods [26], which makes diet a potential confounder of the association between PAH exposures and oxidative stress. In our study, we adjusted for Alternate Mediterranean Diet scores (aMED) as an indicator for antioxidant intake in a subgroup of 125 women for whom we had collected dietary data and found that our results were robust. Maternal employment conditions may also confound the associations, as work-related stress was reported to be associated with urinary concentrations of 8-OHdG in female workers [32, 33]. Our results, however, were robust after adjustment for maternal employment status, possibly because most of the study participants were students or employees of UCLA.

Oxidative stress is one of the main mechanism hypothesized to lead to suboptimal placentation and in turn affect pregnancy success [38]. Our previous study found oxidative stress from air pollution to be associated with adverse birth outcomes [39] as well as with oxidative stress biomarker concentrations in pregnant women [40]. Previous studies have also linked ambient PAH pollutants with adverse birth outcomes [41, 42]. A study of 1,677 women measured urinary PAH metabolites in the 2nd trimester and linked these to preterm birth; female fetuses were found to be more susceptible to 2-PHEN and 1-PYR exposures than the males [7]. Furthermore, a recent study reported sex-modification of associations between urinary OH-PAH concentrations in mid-pregnancy and the placental transcriptome, with more affected transcripts identified in females than males [43].

Oxidative stress has also emerged as one of the underlying mechanism contributing to the toxicity of endocrine disrupting chemicals [44]. In addition, hormonal imbalance leads to compromised antioxidant status and oxidative stress in tissues induces various pathophysiological conditions [45]. Thus, altered hormonal signaling by PAH exposures may contribute to sex differences in oxidative stress levels. A recent cohort study of pregnant women has observed the endocrine disrupting potential of PAH exposures with sex-specific changes in hormone concentrations in pregnancy, which can result in adverse birth outcomes [10, 46]. Oxidative stress pathways may mediate the impact of PAH exposures on adverse birth outcomes via these biological pathways.

Our study indicated that fetal sex may modify the relationship between PAH exposures and oxidative stress during pregnancy, with slightly stronger associations seen for males in early and late pregnancy. Very few studies that investigated PAH exposures and relied on oxidative stress biomarkers among pregnant women examined fetal sex differences, although sex-specific adverse birth outcomes have been well documented, with males more likely to be displaying earlier onset of more severe neonatal complications, which may be due to the sex difference of the placenta [34]. Specifically, male fetuses are more vulnerable to the environmental hazards as they grow faster than females during early placental development [35], which may explain the slightly stronger effect estimates of most PAH exposures on oxidative stress in early pregnancy in our study. Furthermore, PAHs not only can cross the placental barrier, but are also metabolized by the placenta [36, 37]. Thus, our sex-specific associations between PAH and oxidative stress may be indicative of the sex difference in placental response to the PAH exposures. Given the limited sample size and the general overlap of 95% CIs for the sex-specific effect estimates, we cannot exclude the possibility of chance findings. Further studies are necessary to document and understand placental sex differences in response to PAH exposures.

Our study has several strengths. First, the repeated measures in different pregnancy periods made it possible to investigate how the PAH metabolite concentrations throughout pregnancy influence oxidative stress. In addition, repeated urinary measures also made it possible to account for intra-individual variability in oxidative stress biomarkers. Second, the detailed data we collected for environmental and medical covariates enabled sensitivity analyses, such as excluding women more likely to exhibit higher oxidative stress levels such as due to pregnancy disorders. Third, our study population is highly educated and mostly of high socioeconomic status, such that occupational exposures, which are often higher than environmental exposures, are unlikely to have played a role as confounders. Lastly, we used two different oxidative stress biomarkers and evaluated both DNA oxidative damage and lipid peroxidation.

Some limitations need to also be acknowledged. First, the short biological half-live of PAHs limits the informativeness of urinary PAH metabolites as they only reflect recent exposures and to imply that they represent longer term exposures we have to assume relatively constancy of exposures over time [12, 47]. We analyzed urine samples for both the exposure and the outcomes, thus technically using a multiple time point cross-sectional design. Nevertheless, reverse-causation is likely not a concern as PAHs are environmental toxicants that the body metabolizes but does not produce and oxidative stress is a likely consequence, not cause of PAH exposures. Second, even though we collected up to three samples in pregnancy for the participants, our sample size was limited, especially for subgroup analyses. Larger prospective studies with multiple sample collection time points would be helpful to better understand these mechanisms.

## 5 Conclusion

We found that PAH exposures measured with urinary OH-PAH biomarkers are associated with increased oxidative stress generation during pregnancy, which has been previously linked to adverse birth outcomes. We observed potential sex differences that may suggest differential vulnerability during different pregnancy periods for PAH exposures resulting in oxidative stress. Larger investigations are necessary to corroborate these findings.

## Figures and Tables

**Table 1:** Characteristics of the study population (N = 159)

Characteristics	N	%
**Maternal age (years)**		
≤24	3	1.9
25–29	22	13.8
30–34	77	48.4
≥35	57	35.9
**Parity**		
0	74	46.5
≥1	85	53.5
**Maternal race**		
Asian or Pacific islander	45	28.3
Black, non-Hispanic	11	6.9
Hispanic	29	18.2
White, non-Hispanic	73	45.9
American Indian or Alaskan Native	1	0.6
**Maternal education**		
Bachelor’s degree or less	72	46.5
Master’s degree	45	29.0
Doctoral degree or professional degree	38	24.5
Missing	4	
**Employment Status**		
Employed or student	139	89.7
Not employed	16	10.3
Missing	4	
**Pre-pregnancy BMI**		
Underweight	6	3.8
Normal	103	64.8
**Maternal age (years)**		
Overweight	32	20.1
Obese	18	11.3
**Gestational Diabetes**		
Yes	20	12.6
No	139	87.4
**Gestational Hypertension**		
Yes	15	9.4
No	144	90.6
**Pre-eclampsia**		
Yes	16	10.1
No	143	89.9
**Season of Conception**		
Spring	35	22.0
Summer	42	26.4
Fall	36	22.6
Winter	46	28.9

## Data Availability

Data required to reproduce the above findings will be shared via the Human Health Exposure Analysis Resource [HHEAR, formerly Children’s Health Exposure Analysis Resource (CHEAR)] platform maintained by NIEHS contractors.

## Abbreviations

PAH: Polycyclic aromatic hydrocarbons
ROS: reactive oxygen species
8-OHdG: 8-hydroxy-2’-deoxyguanosine
MDA: malondialdehyde
OH-PAHs: hydroxylated metabolites of PAHs
2-NAP: 2-hydroxynapthalene
PARENTs: Imaging Innovations for Placental Assessment in Response to Environmental Exposures study
OH-PAHs: hydroxyl polycyclic aromatic hydrocarbons
CHEAR: Children’s Health Exposure Analysis Resource
2&3-FLUO: combined 2-hydroxyfluorene + 3-hydroxyfluorene metabolites
1-PHEN: 1-hydroxyphenanthrene
2-PHEN: 2-hydroxyphenanthrene
3-PHEN: 3-hydroxyphenanthrene
4-PHEN: 4-hydroxyphenanthrene
1-PYR: 1-hydroxypyrene
Σ4OH-PHEN: sum of 1-, 2-, 3-, and 4- hydroxyphenanthrene
Σ7OH-PAH: sum of combined 2-hydroxy fluorene + 3-hydroxy fluorene metabolites, 2-hydroxynapthalene, 1-hydroxypyrene, 1-hydroxyphenanthrene, 2-hydroxyphenanthrene, 3-hydroxyphenanthrene, and 4-hydroxyphenanthrene
MRI: Magnetic resonance imaging
LOD: limit of detection
SG: specific gravity
BMI: body mass index
aMED: Alternate Mediterranean Diet scores
